# Effect of using preselected markers from imputed whole-genome sequence for genomic prediction in Angus cattle

**DOI:** 10.1186/s12711-025-00999-7

**Published:** 2025-09-25

**Authors:** Nantapong Kamprasert, Hassan Aliloo, Julius H. J. van der Werf, Christian J. Duff, Samuel A. Clark

**Affiliations:** 1 School of Environmental and Rural Science, University of New England, Armidale, NSW 2351 Australia; 2 Angus Australia, Glen Innes Road, Armidale, NSW 2350 Australia

## Abstract

**Background:**

The advent of next-generation sequencing enables the opportunity to use denser marker tools, up to whole-genome sequences (WGS), for genomic prediction in livestock. Improvement in genomic prediction (GP) accuracy from using WGS has been observed in simulation studies. In contrast, such advantage has found to be inconsistent once implemented in practice. The benefit of WGS appears to be from markers that are significant for the trait of interest. Thus, the main objective of this study was to investigate the predictive ability of adding preselected markers to the standard-industry 50k genotype for GP of economically important traits in Angus cattle, namely, birth weight (BW), scrotal circumference (SC), carcass weight (CWT) and carcass intramuscular fat (CIMF). Animals were genotyped with either commercial or customised SNP-genotyping arrays; then, the genotypes were imputed to WGS. The 50k genotype was used as the control group. Informative markers associated with the desired traits were extracted from WGS, then were added to the 50k genotype. Several methods were chosen to select different sets of informative markers, including LD-based pruning, top SNP from a genome-wide association study (GWAS), functional annotation based on Gene Ontology, cattle QTL database, and sequence annotation. In total, eight different sets of genotypes were investigated. We applied different statistical models to predict genomic breeding values, including GBLUP, BayesR, and BayesRC, and two-GRM GBLUP constructed separately from the 50k and the preselected genotype set.

**Results:**

Heritability (h^2^) estimates were similarly calculated using different sets of genotypes and statistical methods across all traits. The log-likelihood ratio values revealed that two-GRM GBLUP was more suitable than the single-GRM GBLUP. There was no significant difference in accuracy and bias among the different sets of genotypes compared to the control group or the statistical methods, except for BW. For BW, the Bayesian models slightly outperformed GBLUP.

**Conclusions:**

The findings suggest that potential improvements may be achieved by using preselected SNPs from the GWAS, a method that has proven within the population. The performance of preselected markers on GP influenced by several factors, including population structure, method used to select significant markers, and genetic architecture of traits.

**Supplementary Information:**

The online version contains supplementary material available at 10.1186/s12711-025-00999-7.

## Background

With the advent of genetic markers, genetic evaluation has evolved to incorporate genomic information through a method known as genomic prediction (GP). Initially, evaluation methods relied on a limited number of genetic markers for evaluation, called marker-assisted selection (MAS). While MAS could partially explain the genetic variance of traits, it faced challenges because the traits of interest in livestock production are polygenic, governed by many genes and the markers associated with those genes. These effects were generally too small to be statistically detected. As a result, the practice of MAS was limited in livestock. Advances in genotyping technologies have enabled the discovery of massive numbers of genetic markers known as single-nucleotide polymorphisms (SNPs), and these types of genetic markers are distributed across the genome. In GP, SNPs are simultaneously incorporated without any significance testing [1]. The assumption is that these SNPs capture the effects of quantitative trait loci (QTL) by capturing the linkage disequilibrium (LD) structure associated with those QTLs. As a result, genomic prediction using SNP genotypes is now common place in animal and plant breeding.

Standard genotype arrays (SNP chips) for most livestock species contain approximately 50,000 SNPs. Developments in genotyping and sequencing have meant that higher density arrays and whole-genome sequences (WGS), are more affordable for livestock species. Hypothetically, a denser SNP chip could provide a more accurate prediction since GP relies on the LD structure between the QTLs and the SNPs. The denser chip raises the probability that every given QTL has a SNP located in perfect LD with it or is the causal variant itself. This would enable GP to operate directly on the causal mutations instead of relying on LD between markers and causative mutations because the causative mutations are expected to be present in the WGS [2].

Improvement in prediction accuracy using WGS has been observed in simulation studies. Meuwissen and Goddard [3] concluded that > 40% increase in the accuracy from WGS-based prediction relative to using 30k-density SNP chips. Clark, Hickey [4] and Iheshiulor, Woolliams [5] reported that a significant increase in prediction accuracy was found when using WGS for oligogenic traits, but prediction accuracy improvements were small for more polygenic or infinitesimal models. Nevertheless, the benefit of WGS in GP has been inconsistent once implemented in practice. The previous study by Zhang, Kemp [6], conducting a study to prove if increasing genotype density can improve prediction accuracy for economic traits in Duroc pigs, concluded that the results varied across traits and were influenced mainly by genetic architecture and statistical methods. However, most studies on direct implementation of WGS in livestock did not demonstrate substantial gain in prediction accuracy compared to the common SNP density. In some cases, the accuracy even decreased when using WGS [7–9]. A possible explanation could be because the range of LD in livestock species is relatively long due to artificial selection [10]. Therefore, the common SNP array may adequately capture the genetic variation, and excessive markers from WGS overparameterized the prediction model.

The benefit of WGS for GP appears to be from markers which are in strong LD with causative variants and significant to the trait of interest. Thus, a subset of the preselected markers can be included to improve GP. Selecting only the predictive markers from a very large number of markers from WGS can also reduce data redundancy of genotypic information, which can cause an overparameterized prediction model. The markers can be curated and selected based on different features, for instance, the association study, functional annotation, and sequence annotation. Cheruiyot, Haile-Mariam [11] and Moghaddar, Khansefid [7] conducted GWAS to select markers from WGS and reported that the preselected markers constantly gained prediction accuracy compared to the standard 50k genotype array. Similarly, Ni, Cavero [12] emphasised that using all markers from WGS did not yield benefit in GP; however, a potential gain in prediction accuracy was suggested when including markers located in the genic region. Lee, Chung [13] concluded that selecting genetic markers based on their biological function only benefited traits affected by a few QTLs with a large effect.

The main objective of this study was to examine the predictive abilities of GP for economically important traits in Angus cattle when adding preselected markers from WGS to the standard-industry 50k SNP array. The traits of interest were birth weight, scrotal circumference, carcass weight and carcass intramuscular fat.

## Methods

### Animals and experimental designs

The phenotypic and genomic data used in this study were obtained from the Angus Australia. Birth weight and scrotal circumference records were from animals in the seedstock and commercial breeders, while two carcass traits were from the Angus Sire Benchmarking Program (ASBP) [14]. The ASBP was established to form a genomic reference for Angus cattle breed. Animals in the ASBP were recorded for routine production traits and hard-to-measure carcass traits (see https://www.angusaustralia.com.au/sire-benchmarking for more details).

### Phenotypes

This study used phenotypic records of four traits associated with body weight, reproduction and carcass (Table 1); namely birth weight (BW, kg), scrotal circumference (SC, cm), and two carcass traits, carcass weight (CWT, kg) and carcass intramuscular fat (CIMF, %). Phenotypic records were, first, edited by removing possible outliers, which were outside four standard deviations from the overall mean. Only animals with both genotypic and phenotypic records were kept in the analysis. BW was measured within 24 h of birth. Range of age for SC, CWT and CIMF were 300 to 700, 400 to 990 and 560 to 990 days, respectively. Other information from the dataset were used to construct fixed effects. Possible fixed effects were tested for their significance, and suitable fixed effects were chosen by Akaike information criterion (AIC). Contemporary group (CG) was included in a statistical model for all the traits studies, following the description outlined in the BREEDPLAN [15]. The CG was a concatenation of herd, year of birth, sex, management group defined by breeders, and measurement date. To ensure comparisons were made among animals in similar environments, the CG effect was further subdivided by age at measurement in 60-day intervals. Records that were duplicated and the CG group of fewer than 40 animals for BW and SC and fewer than 20 animals for CWT and CIMF were excluded from the analysis. Besides the CG effect, additional fixed effects for BW and CWT were linear and quadratic terms of dam age, and linear and quadratic terms of age at measurement for CWT. For SC, further fixed effects were linear and quadratic terms of age at measurement and linear and quadratic terms of BW. While, age at measurement, linear and quadratic terms of dam age, and carcass weight were additional fixed effects for CIMF.

**Table 1:** Summary statistics of phenotypic data in the discovery and prediction sets

Trait	*n*	Mean	SD	Minimum	Maximum
BW	59,039				
Discovery set	19,929	37.00	5.02	23.00	50.00
Prediciton set	39,110	36.23	4.88	23.00	50.00
SC	40,704				
Discovery set	13,352	37.52	2.94	29.00	45.00
Prediciton set	27,352	37.24	3.23	29.00	45.00
CWT	5,499				
Discovery set	2,287	411.26	81.08	200.50	546.40
Prediciton set	3,212	409.62	70.94	211.00	546.00
CIMF	4,251				
Discovery set	1,974	9.86	3.26	3.00	20.50
Prediciton set	2,277	9.49	3.98	3.00	20.50

BW (kg) birth weight, SC (cm) scrotal circumference, CWT (kg) carcass weight, CIMF (%) carcass intramuscular fat

### Genotypes and whole‑genome sequence imputation

Imputed WGS data of Angus cattle were obtained from our previous study [9]. In brief, animals were genotyped with either commercial or customised SNP-genotyping arrays with low to medium density, and the genotypes were imputed to the medium density (50k). A stepwise imputation was performed with the 50k genotypes to obtain WGS. Initially, the 50k genotypes were imputed to high-density genotype, then to the WGS level. Post-imputation quality control removed those SNPs with the inherent imputation accuracy < 0.30 and minor allele frequency (MAF) < 0.0001. The genotypes being analysed for the subsequence step consisted of 44,827 and 7,899,466 SNPs for the 50k and WGS, respectively.

### Cross-validation

For each trait, dataset was split into two non-overlapping sets: (1) a discovery set for preselection of markers using the genome wide association study (GWAS) with imputed WGS and (2) a prediction set for testing a performance of preselected markers to predict genomic breeding values (GEBV). Data splitting was based on year of birth to imitate a forward prediction. The dataset was divided into two subsets independently for each trait due to the difference in the numbers of records between traits and kept the reasonable number of samples for a cross-validation (Table 1). To access prediction robustness and predictive ability, ten-fold cross-validation was performed with the prediction set.

### Preselection of significant markers from whole-genome sequence

The 50k genotype, as an industry-standard genotype, was set as a control group in this study. Predictive markers associated with the desired traits were extracted from WGS and were added to the 50k. In order to select the predictive markers, the traits of interest were grouped by its characteristic; BW was a growth trait, CWT and CIMF were carcass traits, and SC was a reproductive trait. Several approaches were used to select different sets of predictive markers: LD-based pruning, association study between markers and the traits by Genome-wide association study, functional annotation based on the Gene Ontology, QTLs from the Animal QTL Database, and sequence annotation, which are described below. After the marker preselection, each set of preselected markers was pruned for local LD, then clumped with the 50k-genotype SNPs to avoid redundancy of genotype information between the 50k and the preselected markers. The SNP pruning was performed to remove a pair of SNPs in strong LD ( with an R^2^ > 0.95) using PLINK software [16]. Therefore, each treatment consisted of 44,827 SNPs from the 50k genotype and the pruned set of predictive markers from the preselection, except for the LD-based pruning which directly pruned from WGS (Table 2).

**Table 2:** Number of SNPs in each genotype set

Genotypes	Preselcted SNPs	LD pruning within the set	LD clumping with 50k	Preselected SNPs with 50k^1^
50k				44,827
PR1	7,899,466			778,964
PR2				200,000
50kGWAS				
BW	8,987	7,356	6,783	51,610
CWT	8,248	7,709	7,334	52,161
CIMF	8,049	7,491	7,115	51,942
SCR	10,482	8,296	7,679	52,506
50kGO				
Growth	841,818	92,848	85,097	129,924
Carcass	673,292	89,575	84,038	128,865
Reproduction	149,266	18,189	16,321	61,148
50kQTL				
Growth	8,103	3,184	2,282	47,109
Carcass	9,784	4,688	3,797	48,624
Reproduction	6,857	3,575	2,633	47,460
50kSC	53,977	29,778	24,938	69,765
50kSG	2,833,199	364,432	323,093	367,920

*50k*  the common genotype panel set as a control group, *PR1*  pruned WGS, *PR2*  SNPs randomly pick 200,000 SNPs from the pruned WGS, *50kGWAS*  50k with the GWAS outputs, *50kGO*  50k with markers filtered with GO terms, *50kQTL*  50k with the markers from the cattle QTLs, *50kSC*  50k with markers located on the coding region, *50kSG* = 50k with markers located on the genic region

^1^The number of SNPs for genomic prediction

### LD-based pruning

WGS contained 7,899,466 SNPs were pruned based on the local LD to reduce redundancy. SNP pruning for LD was done in PLINK software [16]. The *–indep-pairwise* flag with 5,000 SNPs as the window size in, 100 as the window size step after LD calculation, and 0.95 as the pairwise R^2^ threshold. For each pair of markers with R^2^ > 0.95, the marker with lower MAF was removed. Following the pruning process, a random selection of 200,000 SNPs was made from the pruned WGS dataset to reduce the number of markers further and to randomly select the LD structure associated with genetic markers and QTL.

### Genome-wide association study

GWAS was performed on the discovery set using imputed WGS. The GWAS outputs were utilised to select markers that were associated with the traits of interest. The significant markers below an association threshold of *p*-value 0.01 were referred to as the preselected markers. The association analysis for each trait adopted mixed-model association methods from GCTA software [17] and the model is as follows:

1 $$\mathbf{y}=\mathbf{1}\text{a}+\mathbf{b}\mathbf{x}+\mathbf{g}+\mathbf{e},$$

where **y** is the phenotype, a is the mean term, **1** is a vector of ones, **b** is the fixed effects including the candidate SNP to be tested for association, **x** is the SNP genotype indicator variable coded as 0, 1 or 2, **g** is the polygenic effect as captured by the GRM calculated using all SNPs from imputed WGS using GREML option in GCTA [18] and **e** is the residual.

### Gene Ontology

The Gene Ontology (GO) terms were manually selected based on their biological processes, which were found to be relevant to the traits of interest in the QuickGO [19]. The selection was filtered to include only genes present in *Bos taurus*. For example, GO:0008083 growth factor activity and GO:0048588 developmental cell growth for BW, while GO:0007519 skeletal muscle tissue development and GO:0016459 myosin complex for CWT and CIMF. The GO terms for each trait are available in Additional file 1, Table S1. Also, the GO graphs were constructed to present the association between the terms. Subsequently, the GO terms were annotated utilizing *Ensembl Bos taurus* version ARS-UCD1.3 [20] to identify markers associated with the GO terms for each trait through the *BioMart* [21].

### Cattle QTL database

Cattle QTL database, release 51, was retrieved from the Animal Quantitative Trait Loci Database [22]. The cattle database contained 195,011 QTLs linked to 680 traits relevant to cattle. QTLs were filtered by trait class to match with the traits of interest. The class filter used for BW were “*Production QTL*" and "*Production Association*”, “*Reproduction QTL*” and “*Reproduction Association*” were for SC, and “*Meat and Carcass QTL*" and "*Meat and Carcass Association*” were for CWT and CIMF.

### Sequence annotation

With sequence annotation, WGS were classified into nine categories based on gene-base annotation. The annotation was performed on the ANNOVAR software [23] with the default parameters and used bosTau9 as a reference genome [20]. Our dataset comprised 54,688 SNPs located within coding regions, which included markers associated with exon and splicing regions. The genic-region dataset encompassed all SNPs from the eight classifications: exon, splicing, non-coding RNA (ncRNA), 5' untranslated region (UTR), 3' UTR, intron, upstream, and downstream regions of the genome, resulting in a total of 2,850,201 SNPs identified within the genic region.

### Genomic prediction

Three different statistical methods were used for the prediction of GEBV. These included: GBLUP [24], BayesR [25], and BayesRC [26]. Genomic prediction was performed on the prediction set. The phenotypic data and set of fixed effects for each trait were consistent across both the GBLUP and Bayesian models. In total, we investigated eight different sets of genotypes using different statistical models: (1) the 50k panel as a control group (50k), (2) pruned WGS (PR1), (3) randomly select 200,000 SNPs from pruned WGS (PR2), (4) 50k with the GWAS outputs (50kGWAS), (5) 50k with markers based on GO (50kGO), (6) 50k with the markers derived from the cattle QTLs (50kQTL), (7) 50k with markers located on the coding region (50kSC), and (8) 50k with markers located on the genic region (50kSG).

For each GBLUP analysis, genotypes were fitted in the model with the GRM as a covariance structure of one random additive genetic effect Eq. (2). Besides the additive genetic effect, the random maternal effect was also fitted for BW. In addition to single GRM models, each set of genotypes (50kGWAS, 50kGO, 50kQTL, 50kSC and 50kSG) was fitted as two GRM models with each GRM constructed separately from the 50k and preselected markers genotype set Eq. (3):

2 $$\mathbf{y}=\mathbf{1}\mu +\mathbf{X}\mathbf{b}+{\mathbf{Z}}_{\mathbf{1}}{\mathbf{u}}_{\mathbf{1}}+\mathbf{e},$$

3 $$\mathbf{y}=\mathbf{1}\mu +\mathbf{X}\mathbf{b}+{\mathbf{Z}}_{\mathbf{1}}{\mathbf{u}}_{\mathbf{1}}+{\mathbf{Z}}_{2}{\mathbf{u}}_{\mathbf{2}}+\mathbf{e},$$

Here, $\mathbf{y}$ is a vector of phenotypes; $\mu$ is the intercept; **1** is a vector of ones;$\mathbf{X}$ is an incidence matrix for fixed effects; $\mathbf{b}$ is a vector of fixed effects; $\mathbf{Z}$ is an incidence matrix allocating records to individual additive genetic values in ${\mathbf{u}}_{\mathbf{1}} ({\mathbf{u}}_{\mathbf{1}} \sim \text{N}\left(0,{\mathbf{G}}_{\mathbf{1}}{\upsigma }_{{\text{g}}^{1}}^{2} \right))$, which is a vector of GEBV in which ${\mathbf{G}}_{\mathbf{1}}$ is the GRM formed by different sets of genotypes and ${\upsigma }_{{\text{g}}^{1}}^{2}$ is additive genetic variance; and e is vector of residual effects. While Eq. (3) fitted two components which ${\mathbf{u}}_{\mathbf{1}} \sim \text{ N}\left(0,{\mathbf{G}}_{\mathbf{1}}{\upsigma }_{{\text{g}}^{1}}^{2}\right)$ and ${\mathbf{u}}_{\mathbf{2}}\sim \text{ N}\left(0,{\mathbf{G}}_{\mathbf{2}}{\upsigma }_{{\text{g}}^{2}}^{2}\right)$ are vectors of additive genetic values defied by the SNP sets that constructed ${\mathbf{G}}_{\mathbf{1}}$ and ${\mathbf{G}}_{\mathbf{2}}$ from the 50k and preselected marker genotypes, respectively, ${\upsigma }_{{\text{g}}^{1}}^{2}$ and ${\upsigma }_{{\text{g}}^{2}}^{2}$ are the respective additive genetic variances of those effects. The GEBV from Eq. (3) for each individual was a summary of ${\mathbf{u}}_{\mathbf{1}}$ and${\mathbf{u}}_{\mathbf{2}}$. For BW, the random maternal effect, ${\mathbf{Z}}_{\mathbf{m}}\mathbf{m}$, was also included. The maternal effect was assumed to combine both the maternal genetic and the maternal permanent environmental effect. ${\mathbf{Z}}_{\mathbf{m}}$ is the incidence matrix relating observations to maternal effects, and $\mathbf{m}$ is the maternal effects. We performed GBLUP of GEBV with a univariate animal model on the MTG2 software [27] and genomic residual maximum likelihood (GREML) for estimating variance components and trait heritability. Each GRM was constructed followed Yang, Lee [18] using the GCTA software.

For Bayesian analysis Eq. (4), the genotypes were centred and standardized to a variance of 1. The Bayesian model uses an MCMC approach to estimate SNP effects which are modelled as a mixture distribution of four normal distributions including a null distribution, $\text{N}\left(0, 0\times {\upsigma }_{\text{g}}^{2}\right)$, and three others $\text{N}\left(0, {10}^{-4}\times {\upsigma }_{\text{g}}^{2}\right)$, $\text{N}\left(0, {10}^{-3}\times {\upsigma }_{\text{g}}^{2}\right)$, and $\text{N}\left(0, {10}^{-2}\times {\upsigma }_{\text{g}}^{2}\right)$, where ${\upsigma }_{\text{g}}^{2}$ is the additive genetic variance. The model fitted was:

4 $$\mathbf{y}=\mathbf{1}\mu +\mathbf{X}\mathbf{b}+\mathbf{V}\mathbf{g}+\mathbf{e},$$

where $\mathbf{y}$ is a vector of phenotypes; $\mu$ is the intercept; **1** is a vector of ones;$\mathbf{X}$ is an incidence matrix for fixed effects; $\mathbf{b}$ is a vector of fixed effects; $\mathbf{V}$ is a *n* × *p* matrix of genotypes encoded as 0, 1 or 2 copies of a reference allele;$\mathbf{g}$ is a *p*-dimensional vector of SNP effects; and $\mathbf{e}$ is *n*-dimensional vector of residuals.

The Bayesian models, namely BayesR and BayesRC, were used to predict GEBV for 50kGWAS, 50kGO, 50kQTL, and 50kSC genotype sets, while the 50k set was tested with only the BayesR model. The BayesRC is similar to BayesR except for that the method allows prior biological knowledge for markers by allocating into different categories that may be differentially enriched for QTLs. Thus, in the BayesRC model, the preselected markers were allocated to a separate category from the 50k markers, enabling the use of different mixture distributions of SNP effects for each category. The Bayesian analysis MCMC module ran 20,000 iterations with a 10,000 burn-in period. The GEBV were calculated by multiplying genotypes with corresponding SNP effects summed across the genome. Trait heritability was estimated using the total proportion of phenotypic variance explained by all SNPs.

To assess predictive ability, we conducted a ten-fold cross-validation by randomly dividing individuals into ten groups. The genomic prediction accuracy was calculated as the Pearson correlation coefficient between GEBV and corrected phenotypes, then scaling the correlation by dividing it with the square root of the trait heritability, obtained from the 50k-based analysis. The bias of the prediction accuracy was determined by the regression coefficient of the corrected phenotype on GEBV. Both the prediction accuracy and bias were averaged across each iteration of the cross-validation. Furthermore, the Bayesian heritability, accuracy, and bias were evaluated from each of the five MCMC chains and then averaged.

## Results

### Preselected markers from whole-genome sequence

**Fig. 1:**
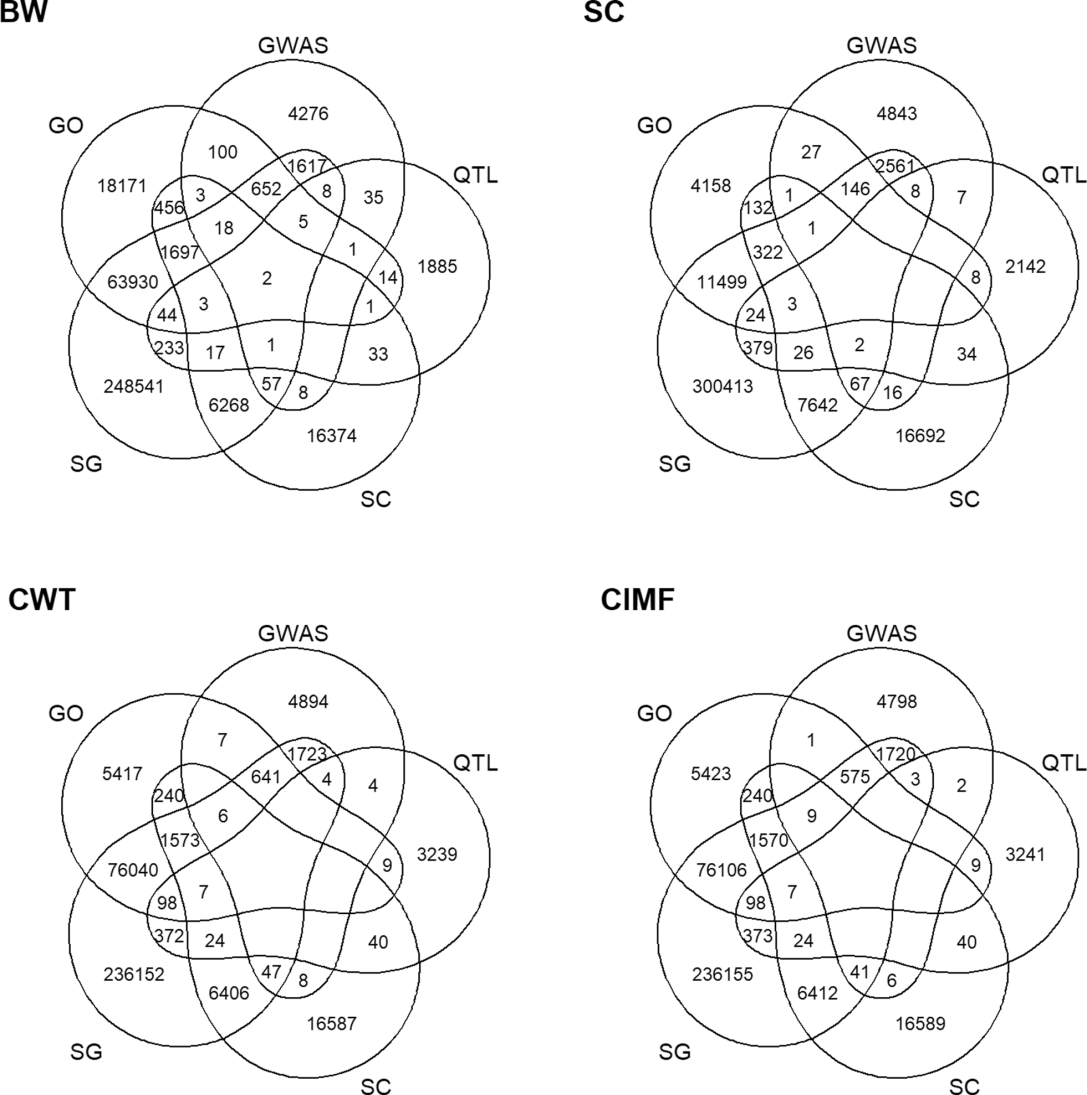
Venn diagram of overlapping preselected markers across different marker preselection methods

The marker preselection was conducted using six different methods, resulting in seven sets of preselected genotypes. Table 2 provides summary of the genotypes for subsequent analysis, including the control and preselected markers from different methods. The last column indicates the number of markers used for genomic prediction, which was based on the 50k panel along with an additional set of preselected markers. Different methods for selecting informative SNPs yielded varying numbers of informative markers. Overlapping preselected markers among five approaches for each trait are illustrated in Fig. 1. The genetic markers derived from WGS were reduced in number through the LD-based pruning, decreasing from 7,899,466 SNPs to 778,964 SNPs. The 50kSG approach provided the largest set of preselected markers, whereas the smallest set was obtained from 50kQTL across all traits. For the GWAS approach, a flexible p-value threshold of 1 × 10^−2^ was applied, as the purpose was to select informative markers rather than to detect QTL. As a result, the GWAS preselected markers were distributed throughout the genome.

### Heritability estimates

The heritability estimates for all traits across a range of scenarios are outlined in Table 3, accompanied by supplementary statistical summaries of variance components, which are provided in Additional file 1, Table S2. The two-GRM GBLUP method facilitated the division of heritability into two components, with the overall trait heritability being derived from the sum of these components. The analyses indicated consistent heritability estimates among different sets of genotypes and statistical models. The significantly higher log-likelihood value suggests that fitting two separate GRMs for GBLUP was preferable compared to including all SNPs into a single GRM. Notably, heritability estimates obtained through Bayesian methods consistently yielded lower values than those derived from the GBLUP method for carcass traits; however, a slightly elevated estimate was observed for BW.

**Table 3:** Heritability estimates for all traits using different set of genotypes and statistical methods

Genotype	Model	BW				SC			CWT			CIMF		
		h^2^_50k_	h^2^_SNP_	h^2^_dam_	h^2^	h^2^_50k_	h^2^_SNP_	h^2^	h^2^_50k_	h^2^_SNP_	h^2^	h^2^_50k_	h^2^_SNP_	h^2^
50k	GBLUP	0.311	–	0.106	0.311	0.347	–	0.347	0.457	–	0.457	0.510	–	0.510
	BayesR	0.329	–	–	0.329	0.339	–	0.339	0.424	–	0.424	0.473	–	0.473
PR1	GBLUP	0.320	–	0.104	0.320	0.354	–	0.354	0.494	–	0.494	0.532	–	0.532
PR2	GBLUP	0.319	–	0.105	0.319	0.353	–	0.353	0.489	–	0.489	0.543	–	0.543
50kGWAS	GBLUP	0.271	–	0.113	0.271	0.313	–	0.313	0.460	–	0.460	0.513	–	0.513
	2-GRM GBLUP	0.141	0.119	0.117	0.260	0.167	0.133	0.299	0.354	0.099	0.453	0.391	0.113	0.504
	BayesR	0.304	–	–	0.304	0.319	–	0.319	0.437	–	0.437	0.476	–	0.476
	BayesRC	0.299	–	–	0.299	0.302	–	0.302	0.425	–	0.425	0.474	–	0.474
50kGO	GBLUP	0.315	–	0.105	0.315	0.349	–	0.349	0.453	–	0.453	0.478	–	0.478
	2-GRM GBLUP	0.237	0.076	0.105	0.313	0.313	0.036	0.348	0.362	0.105	0.467	0.498	0.013	0.512
	BayesR	0.371	–	–	0.371	0.341	–	0.341	0.434	–	0.434	0.439	–	0.439
	BayesRC	0.361	–	–	0.361	0.338	–	0.338	0.441	–	0.441	0.484	–	0.484
50kQTL	GBLUP	0.303	–	0.107	0.303	0.346	–	0.346	0.465	–	0.465	0.518	–	0.518
	2-GRM GBLUP	0.238	0.055	0.110	0.293	0.317	0.031	0.347	0.407	0.056	0.463	0.422	0.098	0.520
	BayesR	0.354	–	–	0.354	0.339	–	0.339	0.437	–	0.437	0.491	–	0.491
	BayesRC	0.342	–	–	0.342	0.334	–	0.334	0.432	–	0.432	0.493	–	0.493
50kSC	GBLUP	0.314	–	0.105	0.314	0.343	–	0.343	0.465	–	0.465	0.516	–	0.516
	2-GRM GBLUP	0.211	0.103	0.105	0.314	0.211	0.133	0.344	0.328	0.137	0.465	0.475	0.039	0.514
	BayesR	0.334	–	–	0.334	0.335	–	0.335	0.445	–	0.445	0.482	–	0.482
	BayesRC	0.349	–	–	0.349	0.332	–	0.332	0.444	–	0.444	0.486	–	0.486
50kSG	GBLUP	0.317	–	0.104	0.317	0.350	–	0.350	0.477	–	0.477	0.523	–	0.523
	2-GRM GBLUP	0.180	0.135	0.105	0.315	0.141	0.210	0.351	0.257	0.222	0.479	0.351	0.177	0.528

The heritability estimates for BW and SC ranged from 0.276 to 0.371 and from 0.299 to 0.354, respectively. Additionally, heritability estimates derived from the 50kGWAS for BW and SC were lower compared to other scenarios, with the lowest estimates for both traits estimating from the 50kGWAS using the two-GRM GBLUP method. For carcass traits, heritability estimates ranged between 0.424 and 0.494 for CWT and between 0.439 and 0.543 for CIMF. The most variability in heritability estimates was noted for CIMF, reflecting a broader range between maximum and minimum values.

### Genomic prediction

**Fig. 2:**
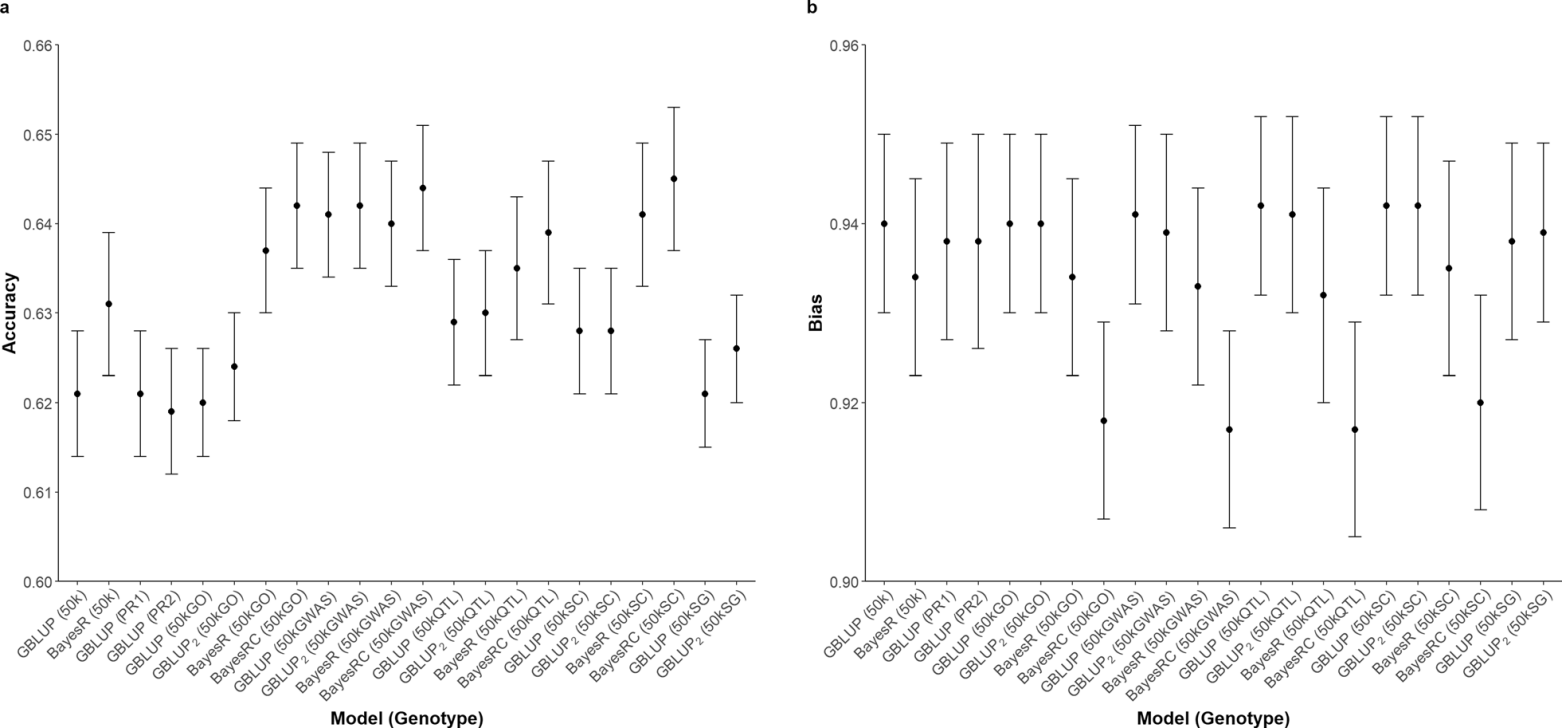
Predictive ability of BW using different sets of genotypes and statistical methods. **a** Prediction accuracy **b** Prediction bias

**Fig. 3:**
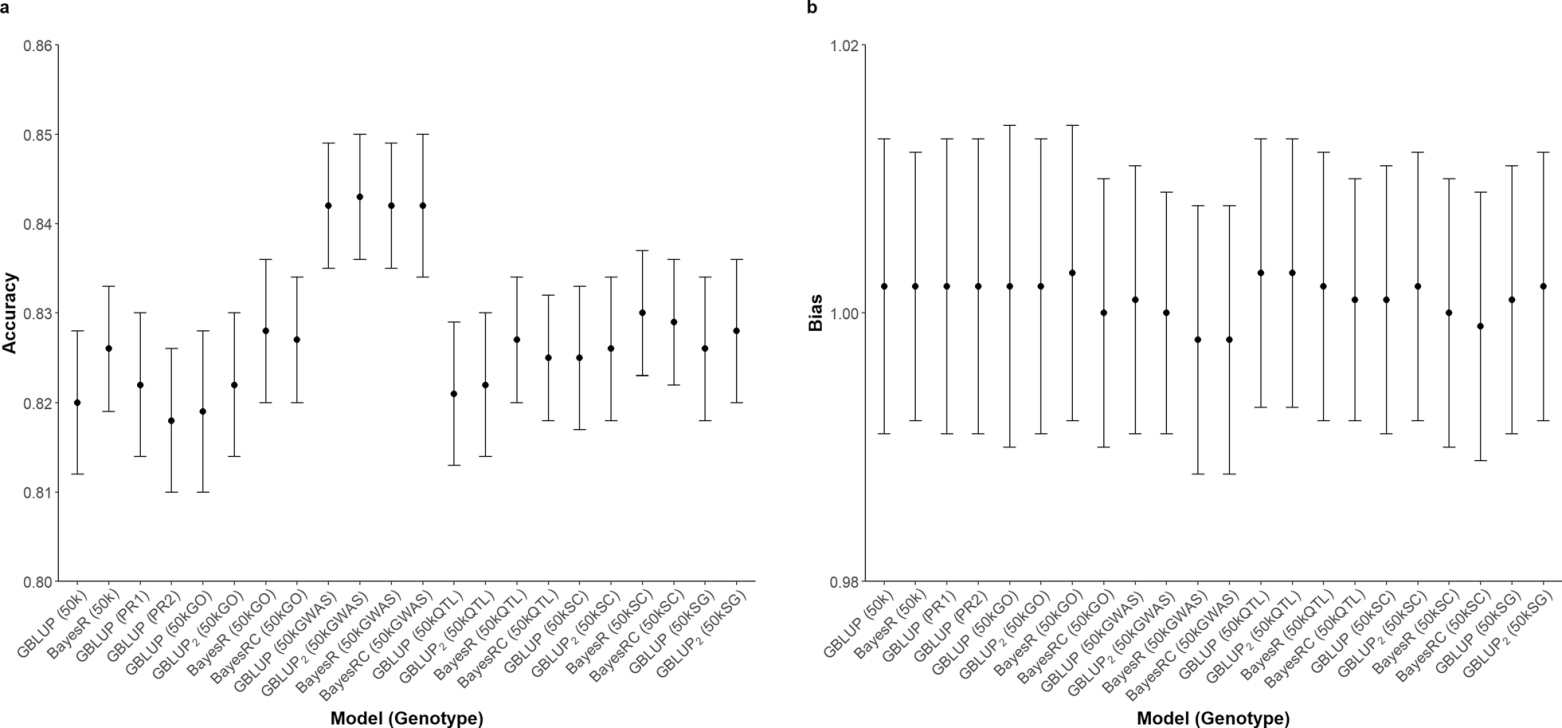
Predictive ability of SC using different sets of genotypes and statistical methods. **a** Prediction accuracy **b** Prediction bias

**Fig. 4:**
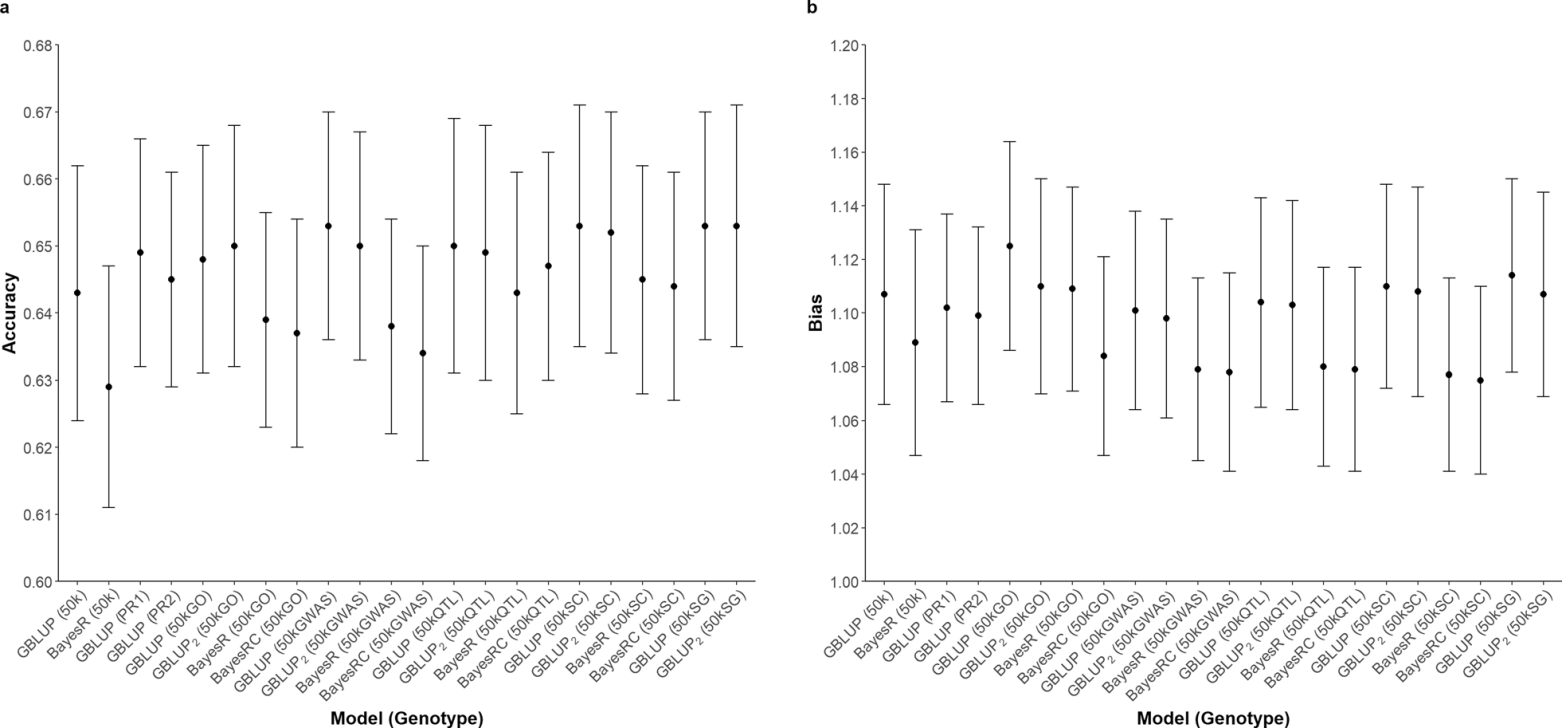
Predictive ability of CWT using different sets of genotypes and statistical methods. **a** Prediction accuracy **b** Prediction bias

**Fig. 5:**
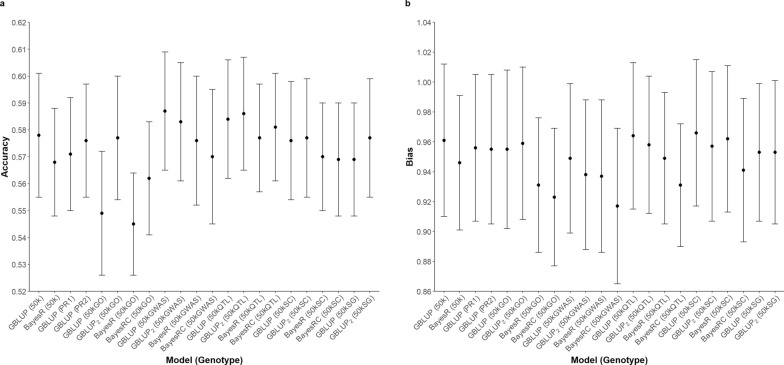
Predictive ability of CIMF using different sets of genotypes and statistical methods. **a** Prediction accuracy **b** Prediction bias

The accuracy and bias of GP across different genotype sets and statistical methods for all traits assessed present in Figs. 2, 3, 4, 5. The prediction accuracy for BW ranged from 0.620 to 0.645, while for SC, it ranged from 0.818 to 0.844. For carcass traits, the accuracy of prediction was between 0.629 and 0.653 for CWT and between 0.545 and 0.587 for CIMF. Although the prediction accuracy varied among different scenarios, these differences were not statistically significant compared to the control group.

The results revealed an inconsistent pattern among the studied traits and highlighted several noteworthy points. When examining statistical methods, both GBLUP and two-GRM GBLUP performed similar levels of prediction accuracy and bias. Bayesian methods predicted with a higher accuracy for BW and SC, with the most substantial improvement observed for BW. Between BayesR and BayesRC, it was observed that BayesRC outperformed BayesR for BW. The Bayesian approach demonstrated its most notable benefit for BW when utilizing the 50kGO dataset, wherein GEBV were found to be overestimated. In contrast, the use of Bayesian methods resulted in decreased prediction accuracy for CWT and CIMF. With regards to CIMF, the prediction accuracy declined by 0.011 with BayesR and by 0.016 with BayesRC when compared to GBLUP using the 50kGWAS dataset.

Improvements in the prediction of GEBV using preselected SNPs were observed in certain instances. A consistent increase in accuracy was noted when incorporating the preselected markers from GWAS across all traits, particularly evident in BW and SC. When using the 50kGWAS, an increase of 0.020 in accuracy was detected for BW and 0.022 for SC, compared to the control group when analysed with GBLUP. Additionally, an increase of 0.010 was reported for the carcass traits with the 50kGWAS. For CWT, similar gains in accuracy were found with the use of preselected markers from the 50kGWAS, 50kSC, and 50kSG. However, a noticeable decline in accuracy was observed for CIMF when using the 50kGO, where the value dropped from 0.578 to 0.549, compared to the control group.

Bias in GP was assessed by examining the regression coefficients of corrected phenotypes against the GEBV across all scenarios (Figs. 2, 3, 4, 5). A deviation of the regression coefficient from one indicated a bias in the prediction. The different sets of genotypes did not show significant differences in bias. Nevertheless, the bias of prediction deviated more from unity with the Bayesian method. This pattern was consistent across the traits studied, particularly evident in BW.

## Discussion

The current study aimed to identify a subset of predictive markers from WGS to evaluate their predictive ability in GP using different statistical methods, each classifying marker based on distinct feature. For example, the LD-based SNP pruning method removes one marker with a lower MAF from each pair of markers that are in strong LD, as these markers are typically co-inherited and provide similar information for prediction. This approach ensures that at least one marker representing each LD block remains across the genome. Additionally, three other approaches utilized publicly accessible databases: Gene Ontology annotation, the Animal QTL Database, and sequence annotation. Each of these approaches concentrated on different feature of the markers. The GO annotation delivers functional insights based on biological processes relevant to the traits studied. The Animal QTL Database aggregated QTL from all publicly available trait mapping data. Sequence annotation regions of interest in DNA sequence, such as post-translational modifications, binding sites, and coding sites. Lastly, GWAS linked genotypes and phenotypes from the same population as the prediction set to identify markers associated with the traits. Each method produced a distinct set of predictive markers, although some markers overlapped between the sets of preselected genotypes. Notably, the 50kSG approach contained the largest number of markers, which were predominantly shared with the 50kGO approach across all studied traits.

A marginally different estimate of heritability was observed across different sets of genotypes, and this variation remained consistent across different traits. Notably, the heritability estimates slightly decreased for BW and SC when using the 50kGWAS. Regarding statistical methods, the results aligned with previous findings by Gurman, Li [28]. The two-GRM GBLUP model, which distinguishes between the genetic markers from the 50k and the predictive set to construct the GRM, yielded significantly higher log-likelihood values. However, heritability estimates derived from the two-GRM GBLUP were generally lower than those obtained from the standard GBLUP. This may be due to the different weighting factors used when constructing the GRM for the two-GRM GBLUP. Meanwhile, the Bayesian method produced slightly higher heritability estimates only for BW.

The findings from BayesR and BayesRC indicated that markers with large effect sizes contributed more to the genetic variance (*V*_*a*_) for BW than to the other traits [See Additional file 2, Figure S1]. This suggests that BW was revealed an oligogenic architecture, whereas the traits of SC, CIMF, and CWT were characterized by a more polygenic structure. Our findings aligned with a previous study by Yin and Konig [29], which suggested that body weights, including BW, in cattle exhibits an oligogenic character, influenced by a few markers with significant effects and many markers with minor effects. However, other studies by Mehrban, Lee [30] and Wang, Zhang [31] reported an oligogenic architecture of carcass traits in cattle, contradicting our results. These contrary observations may reflect the conclusion that the genetic architecture of traits can vary between populations due to distinct genetic histories and selection pressures [32, 33].

The preselection of informative markers was utilized to identify SNPs that affect traits, either by being the causal mutations or within the strong LD with the causal mutations. It was expected that incorporating these preselected SNPs into GP would enhance the accuracy of the prediction, as it would mitigate the limitations associated with the LD structure between SNP markers and causal mutations [34]. However, contrary to this expectation, the current study revealed no substantial improvement in GP regarding the different sets of preselected markers and statistical methods. One possible explanation for this finding could be that the identification of QTL varied across different populations due to their distinct genetic backgrounds. Except for the association study, the marker preselection methods were found using external populations and databases that were not related to the Angus prediction population, which may have led to these preselected markers being less informative for the prediction set. The results aligned with a previous study by Lee, Chung [13], which indicated that a slight benefit was gained by including text-mined SNPs from external sources, and concluded that text mining for marker selection did not take into account the phenotypic or genetic information from a target population. Another study by Ni, Cavero [12] also concurred with the results that a modest gain in predictive ability was observed using the genic region from WGS. Both studies suggested that while no substantial improvement in genomic prediction was noted, deepening our understanding of biological functions, gene networks, pathways, and gene annotations could provide valuable insights for genomic prediction and QTL identification.

Although the results did not show statistical significance, there was an observable potential for improving GP using the GWAS markers. This pattern was particularly evident for BW and SC, while it was less clear for the carcass traits. A possible explanation for this might be the effects of SNPs from the GWA analysis may not have been accurately estimated due to the relatively small dataset available for carcass traits [35].

The observed improvement in GP using significant SNPs from the GWA analysis was consistent with findings from previous studies. For example, a study by Al Kalaldeh, Gibson [36] revealed that prediction accuracy improved by 9% when significant SNPs from GWAS were utilized, compared to the use of common genotypes. Likewise, Moghaddar, Brown [37] confirmed a substantial increase in prediction accuracy with preselected SNPs from GWAS across multiple traits. This potential improvement in the accuracy may be due to the association study, which effectively utilized the information from both the phenotypes and genotypes of same population for the prediction set. As a result, this method effectively identified informative markers that could improve GP.

In addition to identifying a subset of predictive SNPs, it is crucial to evaluate the effectiveness of integrating these SNPs to different genomic prediction models. The GBLUP model with two separate GRMs allows different shrinkage parameters for the set of preselected SNPs, giving them more emphasis in the prediction. This incidence has been observed by Moghaddar, Khansefid [7] and Lee, Chung [13]. Nevertheless, the results, presented in Figs. 2, 3, 4, 5, indicated no significant difference in GP between the single-GRM and two-GRM GBLUP models across all traits.

The Bayesian model, such the BayesR model, employs differentially weighing markers by allocating marker effects to one of the four normal distributions based on statistical evidence in a model selection approach. This approach fits all markers simultaneously, allowing a substantial proportion of markers to have no impact on the studied trait. Similar to the standard BayesR model, the BayesRC model allows the incorporation of user-provided prior knowledge regarding specific groups of variants that may be more enriched for QTL than the others. Within our study, the advantages of Bayesian methods over GBLUP methods were evident only for BW. This may be due to the genetic architecture of traits, as BW was identified as a more oligogenic character compared to the other traits. The findings related to BW align with the literature that a benefit of the Bayesian methods is that the models allow for unequal SNP variances for traits with large-effect QTL [38], also found with high heritability estimates. In contrast, for carcass traits, which exhibited a more polygenic nature, prediction accuracies tended to decline when employing the Bayesian model.

The size of the reference population is generally reported as a key factor that strongly influences predictive ability. Increasing the number of samples in the reference population significantly impacts prediction accuracy, as demonstrated in both simulation and real data studies. The previous studies revealed that prediction accuracy improves with a larger reference population [39, 40]. Thus, using a larger reference population can improve the predictive ability, particularly for the carcass traits.

Although BW had a larger reference set than SC, the prediction ability of SC was more accurate than BW. This could be because the prediction ability, comprising accuracy and bias, is influenced by many factors. In this instance, a possible explanation could be the trait heritability and contemporary group. It has been demonstrated that the more heritable traits would yield more accurate predictions than those with lower heritability [41, 42]. SC, which had higher heritability, resulted in more accurate prediction compared to BW. In addition, predictions based on larger CG sizes have been reported to be more accurate and less biased due to reduced differential effects [43, 44]. An average CG size for SC was higher than BW, with a value of 59.07 and 48.32, respectively.

Another possible explanation for the slight improvement in predictive ability with preselected SNPs from WGS refers to the population structure of the Australian Angus cattle. This population is characterized as purebred commercial cattle that have intensively undergone within-breed selection to achieve substantial genetic progress. The Angus population has small effective population size (*N*_*e*_ = 93) since a relatively small number of founder population [45]. A population with low effective population size is associated with high relatedness between individuals, high extend of LD [46], also low number of segregating chromosome segments [47]. Wientjes, Bijma [32] investigated the long-term consequences of such selective breeding on population structure, revealing that the reduction in genetic variance and the shifts in segregating causal loci change the genetic architecture of traits. This statement aligns with our findings, indicating that the effects of population structure due to the selection are profound, along with the small effective population size. In our study group, the intensity of selective breeding has distinctly shaped its population structure, which affects the selection of predictive SNP, also extend the LD structure. Therefore, the advantages we discovered from employing preselected SNPs in GP stemmed primarily from the within-population method, as opposed to the external subsets, which demonstrated only marginal improvement in prediction accuracy. Our results support the conclusions by Jang, Ros-Freixedes [48], emphasizing that the effectiveness of GP with preselected SNPs was influenced by the population structure and the method employed for marker preselection.

## Conclusions

Subsets of informative markers were obtained from imputed WGS using different methods. These markers were anticipated to enhance predictive ability in GP. Comparisons were made using different statistical models to account for the genetic architecture of the traits. While the statistical differences were not significant, the findings suggest that potential improvements could be achieved by using preselected SNPs from the association study, a method validated within the population, due to the unique genetic background of the population. Additionally, the choice of statistical models was affected by the genetic architecture of traits, where the Bayesian models demonstrating superior performance over the GBLUP models for oligogenic traits. In conclusion, the performance of genomic prediction using preselected SNPs is influenced by multiple factors, including population structure, the method of selecting informative markers, the genetic architecture of the traits, and the statistical models employed.

## Supplementary Information

Additional file 1. Table S1. Gene Ontology terms for the marker preselection. Table S2. Heritability and genetic parameter estimates for all traits using different set of genotypes and statistical methods.

Additional file 2. Figure S1. Proportion of SNPs and SNP effects (*V*_*a*_) in each mixture component from the Bayesian models**.**

## Data Availability

The datasets used and analysed in the current study are available from Angus Australia on reasonable request and with the signing of a material transfer agreement.
