# Supplementary material for: Effect of using preselected markers from imputed whole-genome sequence for genomic prediction in Angus cattle
Source: Genet Sel Evol. 2025 Sep 25;57:52. doi: 10.1186/s12711-025-00999-7 (PMC12465657; doi:10.1186/s12711-025-00999-7)
Supplement: Supplementary file 2 — Additional file 2. Figure S1. Proportion of SNPs and SNP effects (Va) in each mixture component from the Bayesian models. [file 12711_2025_999_MOESM2_ESM.docx]

**
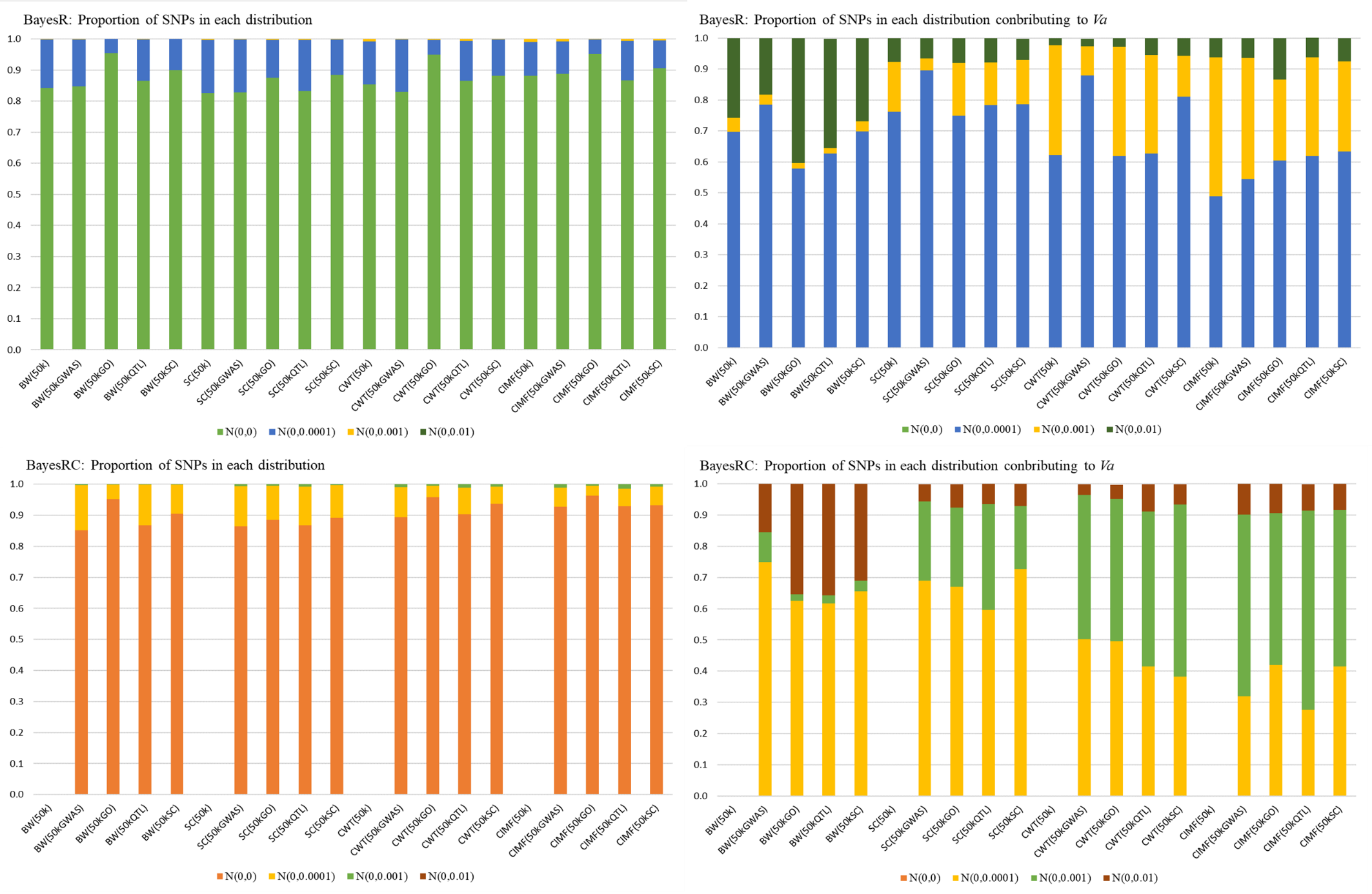
Additional file 2 Figure S1 Proportion of SNPs and SNP effects (*V_a_*) in each mixture component from the Bayesian models**
